# Identification of potential therapeutic antimicrobial peptides against *Acinetobacter baumannii* in a mouse model of pneumonia

**DOI:** 10.1038/s41598-021-86844-5

**Published:** 2021-04-01

**Authors:** Chiau-Jing Jung, You-Di Liao, Chih-Chieh Hsu, Ting-Yu Huang, Yu-Chung Chuang, Jeng-Wei Chen, Yu-Min Kuo, Jean-San Chia

**Affiliations:** 1 Department of Microbiology and Immunology, School of Medicine, College of Medicine, Taipei Medical University, No. 250, Wuxing Street, Taipei, 11031 Taiwan; 2 Institute of Biomedical Sciences, Academia Sinica, Taipei, Taiwan; 3 Graduate Institute of Oral Biology, School of Dentistry, National Taiwan University, Taipei, Taiwan; 4 Graduate Institute of Microbiology, College of Medicine, National Taiwan University, No. 1, Jen Ai Road Section 1, Taipei, 10051 Taiwan; 5 Department of Internal Medicine, National Taiwan University Hospital, Taipei, Taiwan; 6 Division of Cardiovascular Surgery, Department of Surgery, National Taiwan University Hospital, Taipei, Taiwan; 7 Graduate Institute of Clinical Medicine, College of Medicine, National Taiwan University, Taipei, Taiwan; 8 Graduate Institute of Clinical Dentistry, School of Dentistry, National Taiwan University, Taipei, Taiwan

**Keywords:** Antimicrobials, Bacteria

## Abstract

*Acinetobacter baumannii*-induced nosocomial pneumonia has become a serious clinical problem because of high antibiotic resistance rates. Antimicrobial peptides (AMP) are an ideal alternative strategy due to their broad-spectrum of antimicrobial activity and low incidence of bacterial resistance. However, their application is limited by toxicity and stability in vivo. The present study used a mouse model to directly identify potential AMPs effective for treatment of *A. baumannii*-induced pneumonia. Fifty-eight AMPs were screened and two identified (SMAP-29 and TP4) to have prophylactic effects which prevented the death of mice with pneumonia. Furthermore, two TP4 derivatives (dN4 and dC4) were found to have therapeutic activity in pneumonia mouse models by peritoneal or intravenous administration. Both dN4 and dC4 also inhibited and/or eliminated *A. baumannii* biofilms at higher doses. Taken together, these data suggest the AMP derivatives dN4 and dC4 represent a potential treatment strategy for *A. baumannii-*induced pneumonia.

## Introduction

*Acinetobacter baumannii* is a Gram-negative opportunistic pathogen with high incidence among immunocompromised individuals^1^. It largely causes nosocomial and community-acquired pneumonia, skin and urinary tract infections, bacteremia, and surgical site infections^2,3^. The rapid global emergence of multidrug-resistant *A. baumannii* (MDRAB) has been a great public health concern recently^4,5^. The World Health Organization has listed antibiotic-resistant *A. baumannii* as a top priority bacterial pathogen that requires extensive research and development of new and effective antimicrobial agents^6^. In addition, *A. baumannii* is able to form biofilms on the endotracheal surface, which may account for pneumonia onset in patients who require mechanical ventilation^3,7^. These factors substantially contribute to a mortality rate of 35% or higher in patients with nosocomial *A. baumannii* infection^8,9^, depending on the patient’s condition and bacterial strain involved. Therefore, it is imperative to search for new therapeutic approaches to combat the MDR threat from *A. baumannii*.

To treat MDR bacterial infections, various novel antimicrobial strategies have been developed, such as antimicrobial peptides [AMPs]^10^. AMPs are ancient components of the innate immune system found across all kingdoms of life that are less likely to trigger drug resistance^11^. These peptides are composed of a varying number of amino acids (from five to > 100) and usually have a broad spectrum of target organisms, ranging from viruses to parasites. In general, AMPs are cationic, targeting bacterial cell membranes and causing disintegration of the lipid bilayer which leads to cytoplasmic leakage and bacterial death^12^. Other antimicrobial mechanisms of action have also been reported^13^, including delocalization of membrane proteins^14^, alteration of cytoplasmic membrane septum formation^15^, as well as inhibition of cell wall^16^, DNA, RNA, and protein synthesis^17,18^ and enzymatic activity^13,19^. In addition to their bactericidal activity, some AMPs also have the ability to kill bacteria inside the biofilm or inhibit biofilm formation directly^20^. Despite these advantageous features, challenges to the application of AMPs remain, such as potential toxicity to humans^21^, sensitivity to harsh environmental conditions (e.g., extreme pH), and susceptibility to proteases in the circulation^22,23^. Many AMPs work in vitro but fail in vivo. However, because AMPs are composed of amino acids, it is relatively easy to modify their structures in order to lower toxicity and improve stability against proteases^24^. In addition, although previous studies have identified several AMPs exhibiting antimicrobial activity against MDRAB *in vitro*^25,26^, only a few have tested the effects of AMPs in vivo, especially *A. baumannii*-induced pneumonia. Therefore, in the present study, we directly used a mouse model of *A. baumannii*-induced pneumonia to screen for AMPs that can effectively treat MDRAB in vivo. The AMPs having the greatest clinical therapeutic potential were identified in this study.

## Results

### *In vitro* antimicrobial activity of AMPs against laboratory and clinical MDRAB

The antimicrobial activities of 58 AMPs were screened against *A. baumannii* laboratory strains (ATCC strains 17,978 and 19,606) in vitro using a disc diffusion assay (Supplementary Table 1). Among them, 12 exhibited antimicrobial activity: GW-A2, GW-H1a, GW-Q6^27^, bovine myeloid antimicrobial peptide-27 [BMAP-27]^28^, cecropin A (1–8)-melittin (1–10) (CAME)^29^, sheep myeloid antimicrobial peptide [SMAP-29]^30^, latarcin 2a^31,32^, maximin H2^33^, NRC12^34^, pilosulin^35^, pleurocidin^36^, and TP4^37^. Their minimal inhibitory concentration (MIC) and minimal bactericidal concentration (MBC) against *A. baumannii* laboratory and clinical MDRAB strains were further determined (Table 1 and Supplementary Table 2). Notably, most MDRAB strains exhibited resistance to carbapenem antibiotics, including imipenem and meropenem (Supplementary Table 3) and showed susceptibility to these AMPs (Table 1 and Supplementary Table 1).

**Table 1:** Minimal inhibitory concentration (MIC) and minimal bactericidal concentration (MBC) of antimicrobial peptides against *Acinetobacter baumannii* 17,978, 19,606 and multiple drug-resistant (MDR) clinical strains.

Peptide	17,978		19,606		MDRAB clinical strains#	
	MIC (μg/ml)	MBC (μg/ml)	MIC (μg/ml)	MBC (μg/ml)	MIC (μg/ml)	MBC (μg/ml)
A2	32	32	8	8	8–32	8–64
BMAP-27	16	16	8	8	4–16	8–32
CAME	32	32	32	32	8–32	8–64
SMAP-29	8	8	8	8	4–32	4–64
H1a	32	32	16	16	8–16	8–32
Latarcin 2a	16	16	16	16	8–64	8–64
MaximinH2	64	128	32	32	16–128	16–128
NRC12	16	16	16	16	8–32	8–32
Pilosulin	16	32	16	16	8–16	8–32
Pleurocidin	16	16	16	16	8–32	8–32
Q6	16	16	16	16	8–16	8–32
TP4	16	16	16	16	8–32	8–32

^#^Details are available in Supplementary Table 2.

### *In vivo* antimicrobial activity of AMPs in *A. baumannii-*induced murine pneumonia

Murine *A. baumannii*-induced pneumonia models were established by intratracheal inoculation of *A. baumannii* ATCC strain 17978^38^. Three different infection doses were tested: 1 × 10^9^, 5 × 10^8^, and 1 × 10^8^ colony forming unit (CFU)*.* Intratracheal infection of 1 × 10^9^ and 5 × 10^8^ CFU doses resulted in death within 4 d (Supplementary Fig. S1A). Although the 1 × 10^8^ CFU dose did not cause death, *A. baumannii* was detected in the lung tissue along with increased infiltration of neutrophils and macrophages (Supplementary Fig. S1B,C,D).

**Figure 1:**
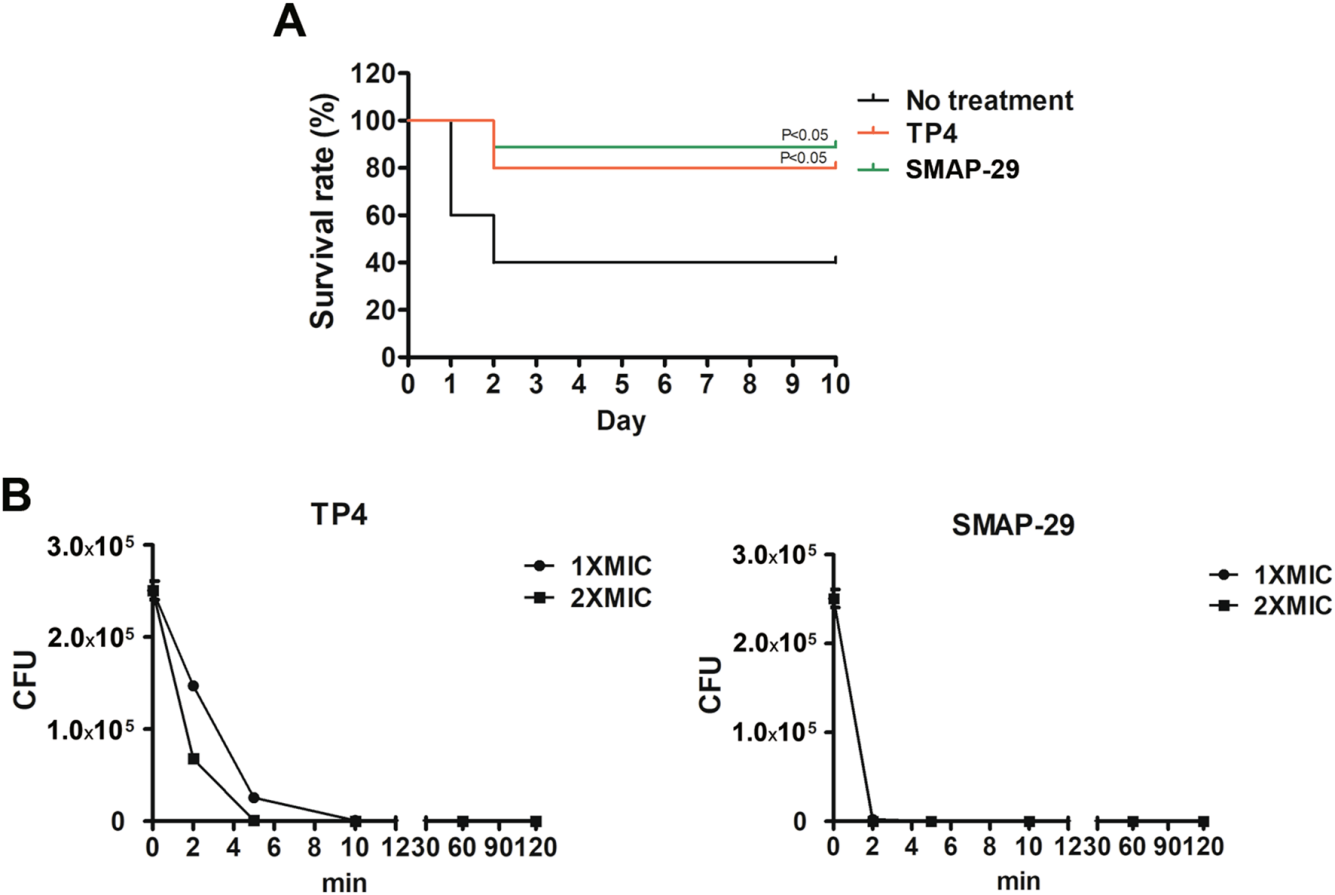
Prophylactic intratracheal administration of TP4 and SMAP-29 reduced mortality associated with *A. baumannii*-induced pneumonia. (**A**) Mice were intratracheally infected with *A. baumannii* ATCC 17,978 at a dose of 5 × 10^8^ CFU. A 10-μL aliquot of 2 × MIC of TP4 (16 μg/mL) and SMAP-29 (32 μg/mL) was intratracheally administered 30 min before bacterial infection and mouse survival detected daily (*n* = 10/group). (**B**) *A. baumannii* ATCC 17,978 (2–5 × 10^5^ CFU) were mixed with 1 × and 2 × MIC of TP4 (8 and 16 μg/mL) or SMAP-29 (16 and 32 μg/mL) and incubated at 37 °C. The survival bacteria were detected by plating on agar plate.

To evaluate the effect of AMPs, an infection dose of 5 × 10^8^ CFU was used and mouse survival monitored. Since studies have indicated AMPs have cellular toxicity or lose antimicrobial activity in the circulation, the prophylactic effect of prescreened AMPs in vivo was examined by intratracheal administration in pneumonia mouse models. Among the 12 AMPs tested, only SMAP-29 and TP4 significantly prevented the mortality of *A. baumannii-*infected mice (Fig. 1A). Notably, the results of the in vitro time killing assay showed that SMAP-29 and TP4 can rapidly kill *A. baumannii* within 10 min (Fig. 1B).

### *In vitro* antimicrobial activity of TP4 derivatives against laboratory and clinical MDRAB

SMAP-29 and TP4 have high potential for controlling microbial infections^39,40^. Both exhibit broad-spectrum activity against bacteria and show therapeutic activity in mice. However, their high cellular toxicity and hemolytic activity limit their application. Previous studies have enhanced the application potential of SMAP-29 by modifying its structure^41,42^. Similarly, the hemolytic activity of TP4 was reduced in our previous study by generating truncated and amino acid-substituted analogues^43^. The antimicrobial activities of these derivatives against *Pseudomonas aeruginosa*, *Staphylococcus aureus*, and *Candida albicans* were also investigated in the same previous study^43^. Here, the antimicrobial activity (MIC and MBC) of TP4 derivatives (dN2, dN4, dC2, dC4, F1A/I2A, H3A/H4A, I5A/I6A, L9A/F10A, I16R, I16E, I16A, L19H/I20H, A12V/A15H, A12I/A15I, R18S/R21H, VHSH, and TP3) was tested in vitro against laboratory and clinical MDRAB. Most of the TP4 derivatives that exhibited reduced hemolytic activity^43^ also showed antimicrobial activity but higher MIC/MBC level against MDRAB strains in vitro (Table 2).

**Table 2:** Minimal inhibitory concentration (MIC) and minimal bactericidal concentration (MBC) of antimicrobial peptide TP4 derivatives against *Acinetobacter baumannii* ATCC and clinical multiple drug-resistant (MDR) strains.

Peptide	ATCC strains				MDRAB strains			
	17,978		19,606		BF50		MO91	
	MIC (μg/ml)	MBC (μg/ml)	MIC (μg/ml)	MBC (μg/ml)	MIC (μg/ml)	MBC (μg/ml)	MIC (μg/ml)	MBC (μg/ml)
TP4	16	16	16	16	16	16	16	32
dN2	32	32	64	64	32	64	64	64
dN4	32	32	16	64	16	64	32	32
dC2	16	16	16	32	16	32	16	32
dC4	32	32	32	32	32	32	32	32
F1A/I2A	32	32	16	16	16	32	32	32
H3A/H4A	64	128	64	64	64	128	64	256
I5A/I6A	32	32	64	64	32	64	64	128
L9A/F10A	64	64	64	128	64	64	128	128
I16R	128	128	64	64	64	128	64	256
I16E	> 256	> 256	> 256	> 256	> 256	> 256	> 256	> 256
I16A	16	32	32	32	16	16	16	32
L19H/I20H	32	32	64	64	32	32	32	32
A12V/A15H	16	32	16	32	16	128	16	256
A12I/A15I	64	64	32	128	64	128	32	128
R18S/R21H	16	32	32	32	16	64	32	32
VHSH	16	64	16	16	32	32	32	64
TP3	32	32	16	32	32	32	16	32

### *In vivo* therapeutic effect of TP4 derivatives in *A. baumannii*-induced murine pneumonia

**Figure 2:**
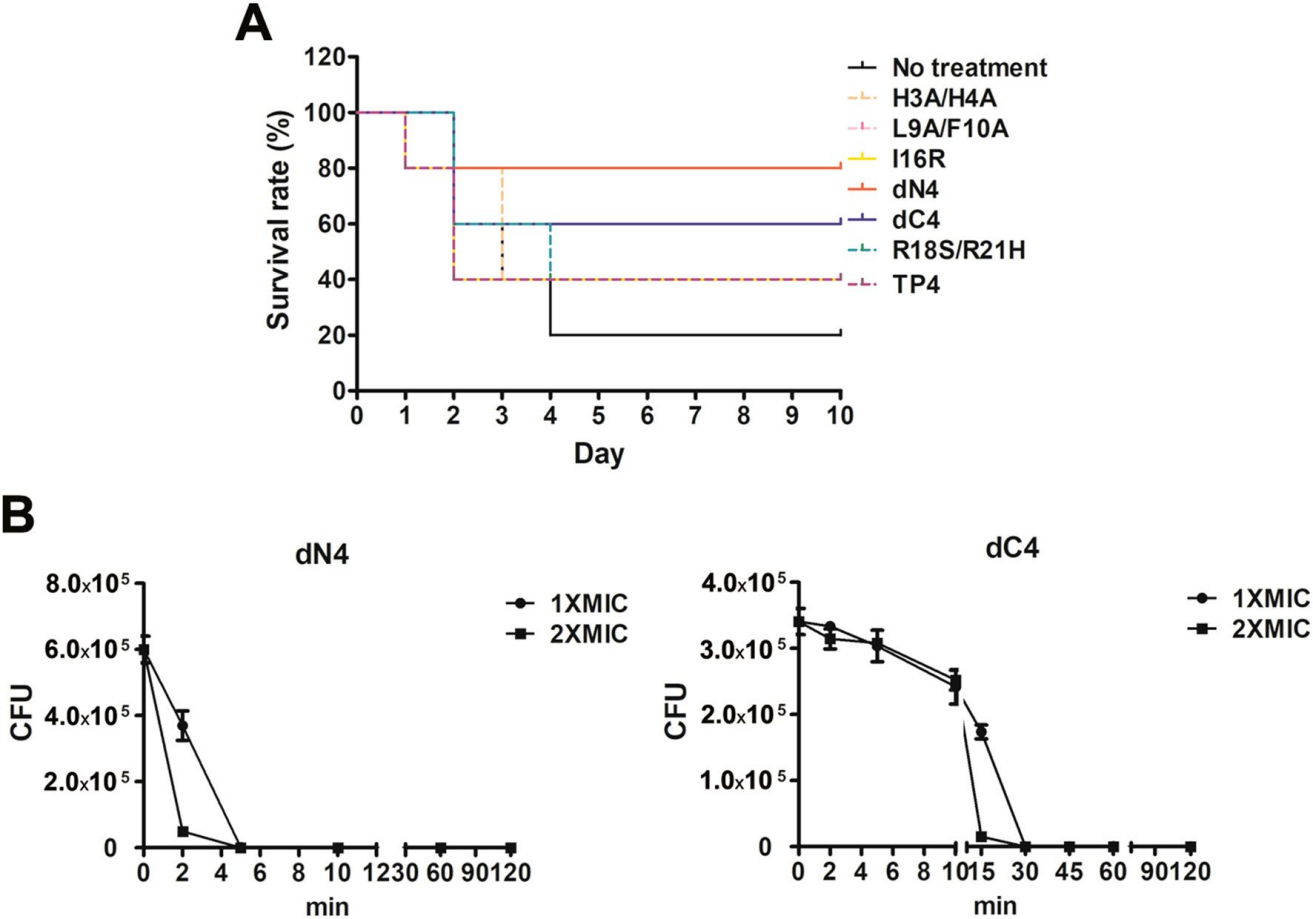
Peritoneal administration of TP4 derivatives in *A. baumannii*-induced pneumonia mouse models. (**A**) Mice were intratracheally infected with *A. baumannii* ATCC 17,978 at a dose of 5 × 10^8^ CFU. TP4 derivatives (2 mg/kg) were peritoneally administrated 4 h after bacterial infection, and mouse survival was detected daily (*n* = 5/group). (**B**) *A. baumannii* ATCC 17,978 (2–5 × 10^5^ CFU) were mixed with 1 × and 2 × MIC of dN4 (32 and 64 μg/mL) or dC4 (32 and 64 μg/mL), and incubated at 37 °C. The survival bacteria were detected by plating on agar plate.

**Figure 3:**
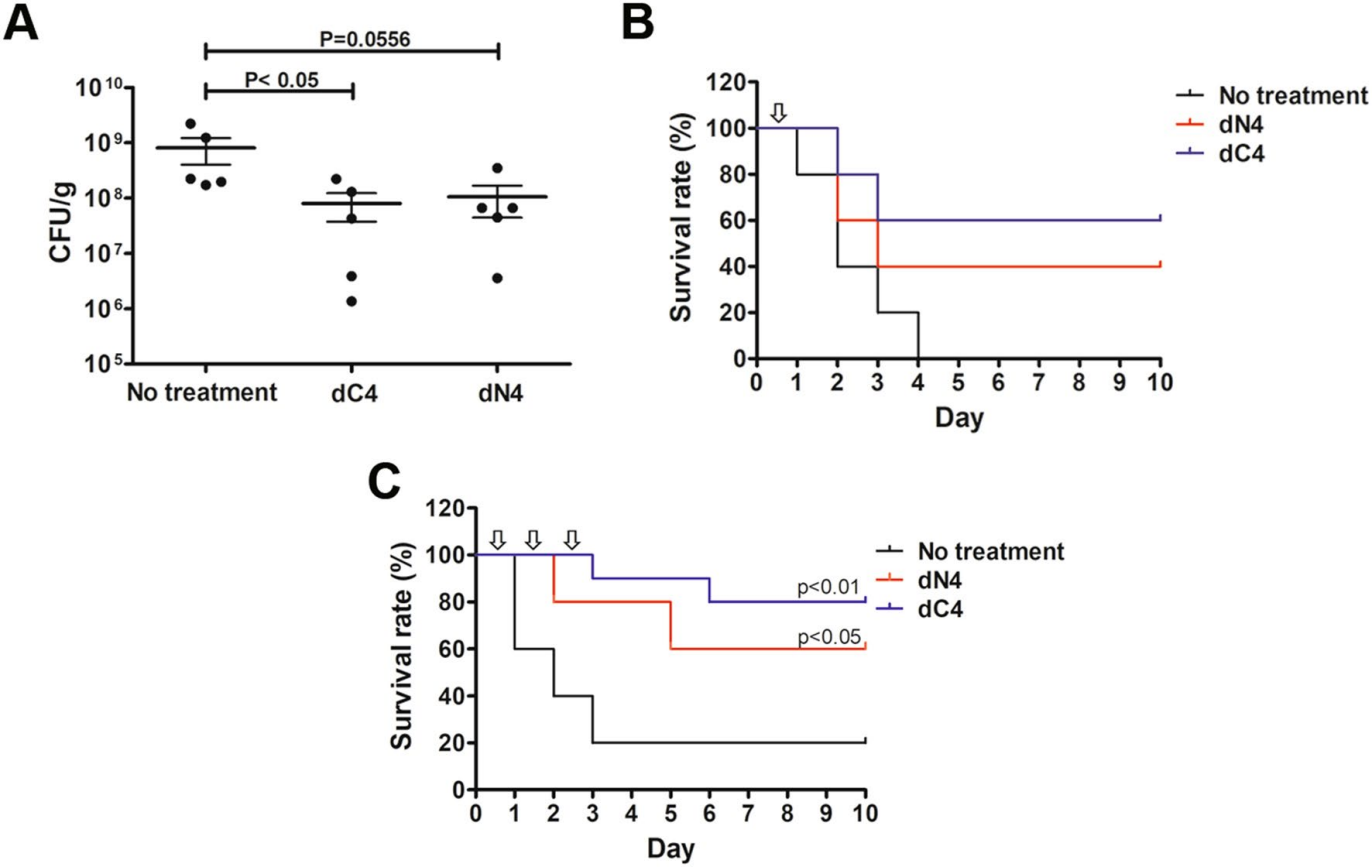
Intravascular administration of dN4 and dC4 reduced bacteria numbers in lung tissue and reduced mortality associated with *A. baumannii-*induced pneumonia. (**A**) Mice were intratracheally infected with *A. baumannii* ATCC 17,978 at a dose of 5 × 10^8^ CFU. dC4 or dN4 (2 mg/kg) was intravascularly administrated 4 h after bacterial infection. Infected mice were sacrificed 1 d after infection, and lung tissue was isolated. Bacteria that colonized lung tissue were quantified by plating on LB agar plates. Each point represents one mouse. Data represent means ± standard error of the means and were statistically analyzed using Mann–Whitney U-test versus the no treatment group. (**B**) Mice were intratracheally infected with *A. baumannii* ATCC 17,978 at a dose of 5 × 10^8^ CFU. dC4 or dN4 (2 mg/kg) was intravascularly administrated 4 h after bacterial infection. Mouse survival was detected daily (*n* = 5/group). (**C**) Mice were intratracheally infected with *A. baumannii* ATCC 17,978 at a dose of 5 × 10^8^ CFU. dC4 or dN4 (2 mg/kg) was intravascularly administered once a day for 3 d. Mouse survival was detected daily (*n* = 10/group).

Peritoneal administration of TP4 reportedly exhibits a therapeutic effect in *S. aureus*-induced infection in mice^39^. Therefore, the therapeutic effect of peritoneal administration of TP4 derivatives in *A. baumannii*-induced pneumonia mouse models was assessed in the present study. Among 17 TP4 derivatives, six (dN4, dC4, H3A/H4A, L9A/F10A, I16R, and R18S/R21H) enhanced the survival rate of pneumonia mouse models (Fig. 2). Notably, dN4 and dC4 enhanced survival the most and had the lowest hemolytic activity^43^. In addition, dN4 and dC4 can also rapidly kill bacteria (Fig. 2B). Therefore, the therapeutic effect of intravenous dN4 and dC4 in mice was examined (Fig. 3). Intravenous administration of dN4 or dC4 4 h after inoculation of mice with *A. baumannii* reduced bacterial numbers inside the lung and enhanced the survival rate (Fig. 3A,B). Administration of dN4 or dC4 once a day for three days enhanced more mouse survival to chi-square significantly enhanced level (Fig. 3C). These data suggest the potential of dN4 and dC4 in the clinical control of *A. baumannii*-induced pneumonia.

### dN4 and dC4 exhibited antimicrobial activity against *A. baumannii* biofilm

**Figure 4:**
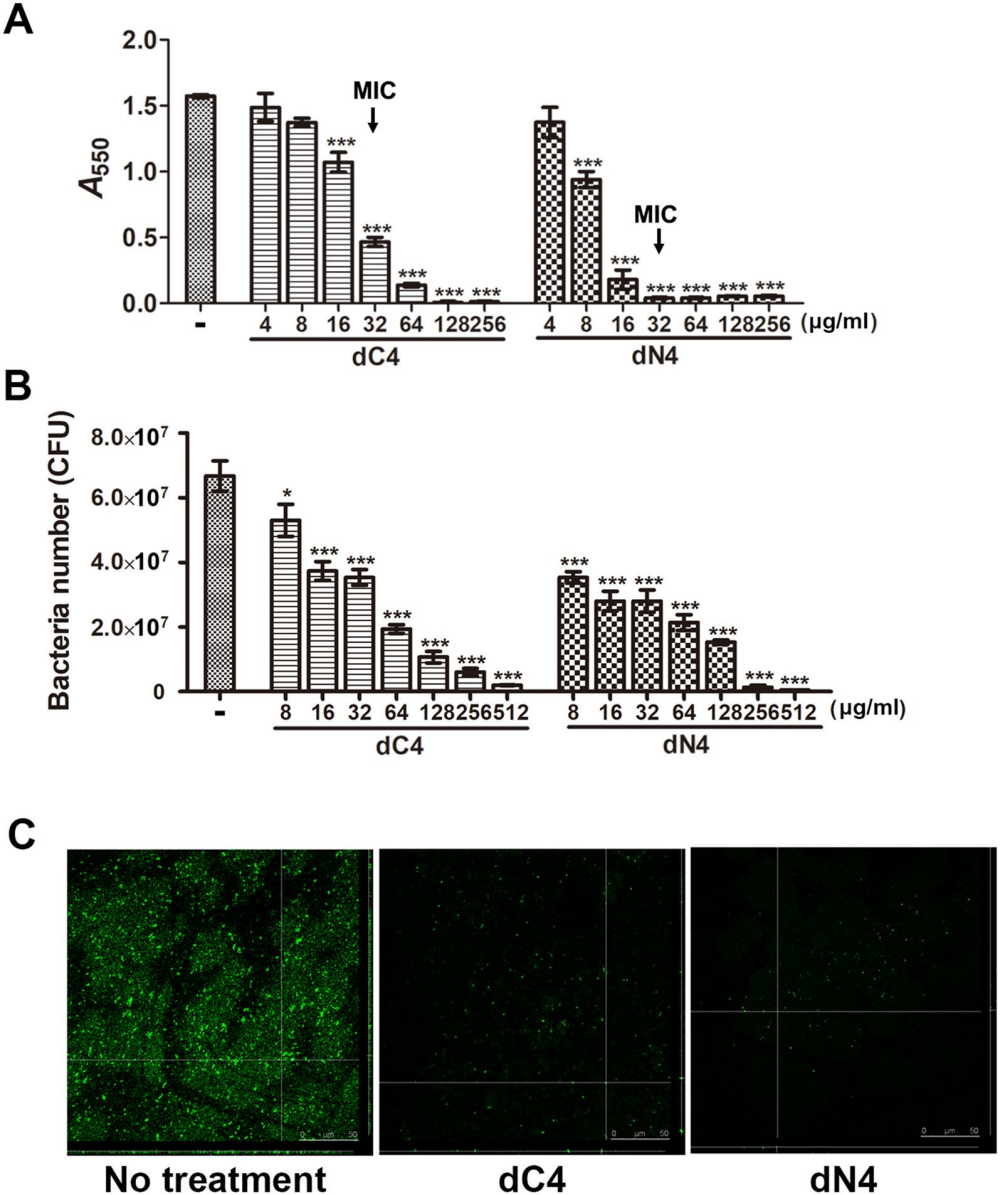
dC4 and dN4 inhibit formation of and eliminate established *A. baumannii* biofilm. (**A**) dC4 and dN4 inhibition of *A. baumannii* biofilm formation. *A. baumannii* biofilm was grown in LB medium containing serial concentrations of dC4 or dN4 and stained with 0.1% crystal violet. Staining was detected by measuring the absorbance at 550 nm. Data are expressed as the means ± standard deviations of triplicate experiments; ***P < 0.001 by 1-way analysis of variance. (**B**) dC4 and dN4 elimination of *A. baumannii* biofilm. LB medium containing serial concentrations of dC4 or dN4 was added onto formed *A. baumannii* biofilm. After overnight incubation, the survived bacteria were quantified by plating on LB agar plates. Data are expressed as means ± standard deviations of triplicate experiments; ***P < 0.001 by 1-way analysis of variance. (**C**) Confocal laser scanning microscopy images show dC4 and dN4 elimination of *A. baumannii* biofilm. LB medium with or without 256 μg/mL of dC4 or dN4 was added to established *A. baumannii* biofilm. *A. baumannii* were labeled with GFP (green). After overnight incubation, the biofilms were observed by confocal microscope (400 × magnification).

In addition to colonizing lung tissue, *A. baumannii* can form a biofilm on the surface of the endotracheal tube which may account for causing pneumonia in patients who require mechanical ventilation^3,7^. Therefore, the antimicrobial activity of dN4 and dC4 against *A. baumannii* biofilm were further tested. Both of dN4 and dC4 inhibited *A. baumannii* biofilm formation in a dose-dependent manner (Fig. 4A) and eliminated established biofilm (Fig. 4B,C). These data suggest that both dN4 and dC4 can prevent and/or eliminate *A. baumannii* biofilm on the surface of the endotracheal tube.

## Discussion

MDR bacteria-induced infection is one of the toughest issues facing clinics today as several are currently untreatable, including MDRAB*.* Among alternative strategies under development, AMPs are ideal antimicrobial agents because they do not readily trigger resistance. However, application of AMPs is limited by their high cellular toxicity and loss of function in the circulation^23^; hence, many AMPs work in vitro but fail in vivo. Therefore, the present study identified AMPs exhibiting potential to treat *A. baumannii-*induced pneumonia in mice. In particular, intravenous adminstration of TP4 derivatives dC4 or dN4 reduced *A. baumannii* conlonization in the lung and enhanced survival, suggesting preservation of antimicrobial activity in the circulation. In fact, dN4 and dC4 maintained, though reduced, antimicrobial activity against *A. baumannii* in the presence of plasma components in vitro (Supplementary Table 4), implying these derivatives may be more resistant to proteases in the plasma relative to other AMPs tested. In addition, our preliminary data showed that the LD_50_ of SMAP-29 and TP4 is around 10–20 mg/kg by intratracheal administration and the LD_50_ of dN4 and dC4 is approxmiately 80–90 mg/kg and 90–100 mg/kg, respectively, by intravenous administration. However, additional large scale animal testing is needed to determine the cellular toxicity and stablity/half-life of dN4 and dC4 for pharmaceutical applications. Although intravenous administration of dN4 or dC4 enhanced survival, it should be noted that this required daily administration for three days to achieve significant results. On the other hand, the sample number was small as this was a pilot study conducted to identify AMPs with the potential to treat MDRAB in vivo. More studies with a larger sample size and more dose testing should be performed to confirm the clinical potential of dN4 and dC4.

We previously showed that TP4 exhibits broad-spectrum activity against various bacteria, including *P. aeruginosa*^43^*.* TP4 binds the *P. aeruginosa* outer membrane target protein OprI through hydrophobic interactions. Mutation of hydrophobic residues in the main helix or deletion of nonstructural coils at N- and C-termini significantly reduced the hemolytic activity of TP4 and reduced the antimicrobial activity against *P. aeruginosa*^43^. The current results showed that most TP4 derivatives, which showed reduced hemolytic activity and reduced antimicrobial activity against *P. aeruginosa*^43^, maintained their antimicrobial activity against *A. baumannii* in the present study (Table 2). This suggests the antimicrobial mechanisms of TP4 against *A. baumannii* and *P. aeruginosa* are different*.* Although TP4 and many of its derivatives have in vitro antimicrobial activity against *A. baumannii*, only dN4 and dC4 showed clear protection against *A. baumannii*-induced pneumoniae in vivo (Fig. 2). This may be attributed to the significantly reduced hemolytic activity of dN4 and dC4 compared with TP4 and other TP4 derivatives^43^. In addition, dN4 and dC4 showed comparatively low antimicrobial activity (32 μg/mL) and low in vivo bactericidal effects (Fig. 3A) with a relatively low therapeutic dose, indicating an indirect antimicrobial effect of dN4 and dC4 in vivo. Moreover, our preliminary data showed that intratracheal administration of dN4 or dC4 can only delay death and not enhance survival, which also supports the indirect antimicrobial activity of dN4 and dC4 in the circulation. TP4 has been shown to modulate the host immune response thereby enhancing clearance of *Helicobacter pylori* infection^44^. Therefore, the indirect antimicrobial effect of dN4 and dC4 in vivo may be attributed to the immune modulation ability of these AMPs against *A. baumannii* infection, which will require further elucidation.

In addition to colonizing lung tissue, *A. baumannii* form a biofilm on the surface of the endotracheal tube, accounting for pneumonia onset in patients requiring mechanical ventilation^3,7^. Biofilm-producing *A. baumannii* clinical isolates have also been shown to be more resistant to most antibiotics compared with non-biofilm-producing isolates^45^. Bacterial biofilm is well-organized and dense so as to support bacteria localization and growth on a specific surface and facilitate escape from immune surveillance and antimicrobial molecule attack^46^. Therefore, bacterial biofilms are even more resistant to antibiotic treatment versus platonic populations. Although the current results showed that the *A. baumannii* biofilm is more resistant to dN4 and dC4 than platonic populations, both derivatives can successfully inhibit *A. baumannii* biofilm formation in vitro (Fig. 4A) as well as eliminate established biofilms and the bacteria residing in them (Fig. 4B,C). These results suggest that dN4 and dC4 may be particularly useful for endotracheal tube-associated *A. baumannii* infections. However, the dose of 256 μg/mL was shown to eliminate almost the bacteria inside the biofilm, which is a little high for clinical use. Therefore, use of dN4 and dC4 to eradicate established biofilms on an endotracheal tube will require further evaluation.

In summary, the present study identified two AMPs (SMAP-29 and TP4) that exhibited in vitro and in vivo prophylactic effects against *A. baumannii.* Furthermore, TP4 derivatives dC4 and dN4 exhibiting reduced hemolytic activity but high antimicrobial activity against *A. baumannii* both in vitro and in vivo (intravenously and peritoneally). In addition, our preliminary data showed dN4 and dC4 also have antimicrobial activity against other bacteria, including antibiotic-resistant pathogens. Considering the extremely limited strategies available to treat MDR pathogen-induced infections, the clinical potential of these AMPs in other MDR pathogen-induced infections should be further evaluated.

## Materials and methods

### Bacterial strains and growth conditions

*A. baumannii* laboratory strains (ATCC 17,978 and 19,606) and clinical isolates from National Taiwan University Hospital (Taipei City, Taiwan) were grown and maintained in Luria–Bertani (LB) broth and agar plates. Medium containing 50 μg/mL kanamycin was used to propagate *A. baumannii* carrying plasmids that expressed green fluorescent protein (GFPuv).

### MIC and MBC of AMPs

All methods used in this study were carried out in accordance with relevant guidelines and regulations.

All AMPs and TP4 derivatives used in this study were synthesized by Kelowna International Scientific Inc. (Taipei City, Taiwan) with more than 95% purity, and their molecular weights were verified by mass spectrum analysis. MBC were determined by extending the MIC procedure in sterilized 96-well polypropylene microtiter plates according to the broth microdilution guidelines of the Clinical and Laboratory Standards Institute with some modification^47^. Briefly, 5 × 10^5^ CFU/mL of bacteria were inoculated in LB medium containing AMPs or antibiotic at final concentrations ranging from 512 to 1 μg/mL (from serial twofold dilutions). After incubation for 24 h at 37 °C, the lowest concentration showing no visible growth was used as the MIC value. In addition, 10 µL was drawn from each well with no visible turbidity onto LB agar plates and incubated for 24 h at 37 °C. The lowest concentration showing no visible growth on agar subculture was used as the MBC value. All experiments were performed in triplicate.

### Experimental *A. baumannii*-induced pneumonia mouse model

Approval for animal use was obtained from the National Taiwan University Institutional Animal Care and Use Committee prior to initiation of experiments. The study was carried out in compliance with the ARRIVE guidelines (https://arriveguidelines.org). The experimental *A. baumannii*-induced pneumonia mouse model was generated as previously described with some modification^38^. Male BALB/c mice that were 6–8 weeks-old were purchased from the National Laboratory Animal Center (Nangang Dist, Taipei City, Taiwan). Briefly, 5 × 10^8^ CFU bacteria were intratracheally inoculated into mice and the survival rate was monitored daily for 10 d. To evaluate prophylactic effects of AMPs, 10 μL of 2 × MIC of each AMP was intratracheally administrated to mice 30 min before inoculation of bacteria. For evaluating the therapeutic effect of TP4 derivatives, 2 mg/kg of each derivative was peritoneally or intravenously administrated 4 h after bacterial infection or intravenously administrated once a day for three days.

To monitor bacterial colocalization in the lung, mice were sacrificed 1 d after bacterial infection. Dissected lung tissue was homogenized in a sterile disposable tissue grinder, serially diluted in phosphate-buffered saline (PBS), and plated on LB agar to determine the CFU/g of lung tissue. For histologic analysis, lung tissue was fixed with 2% paraformaldehyde, embedded in paraffin, cut into 3-μm-thick slices, and stained with hematoxylin and eosin. Immunohistochemistry was performed using primary antibodies against Ly6G (ab21595; Abcam, Cambridge, MA, USA) or F4/80 and developed with diaminobenzidine (Dako, Santa Clara, CA, USA) followed by counterstaining with Mayer’s hematoxylin.

### Construction of GFP-tagged *A. baumannii*

For construction of GFPuv-tagged *A. baumannii*, an *A. baumannii* GFPuv-expressing plasmid was generated. Briefly, an *A. baumannii* promoter PgroEL from *groEL* of *A. baumannii* was amplified from *A. baumannii* chromosomal DNA using primers AB_groELPF_XbaI (5′ CGATTATCTAGAGGTGAAGAACTCTTAATTATG-3′) and AB_groELPR_KpnI (5′-CGATTAGGTACCCTTGAAACTTCACGAACAAG-3′). The polymerase chain reaction (PCR) product was digested with KpnI and XbaI and inserted upstream of the gene sequence of GFPuv in the pGFPuv plasmid (Clontech Laboratories, Inc., Mountain View, CA). The GFPuv coding sequence containing the promoter PgroEL was further PCR-amplified using primers AB_groELPF_XbaI and GFPR_XbaI (5′-CGA TTA TCT AGA TTA TTT GTA GAG CTC ATC CAT-3′). The PCR product was digested with XbaI and inserted into the *E. coli*-*A. baumannii* shuttle plasmid pAbYm3. The resulting plasmid, pABYm3-GFPuv, was transformed into *A. baumannii*, and positive clones were selected by green fluorescence following ultraviolet excitation (365 nm).

### Biofilm formation assay

Bacterial biofilm assay was performed as previous described^48^ with modification. Briefly, approximately 10^7^ CFU bacteria in 200 μL of LB medium was added to individual wells of a 96-well polystyrene microtiter plate. To investigate the inhibitory effect of AMPs on biofilm formation, serial concentrations of AMPs were added to the culture medium when inoculating bacteria. After a 16-h incubation at 37 °C, the medium was removed, and biofilms were detected by crystal violet staining assay^48^. Each assay was performed in triplicate, and wells without biofilms were used as blank controls. To investigate the effect of AMPs on established biofilm, the biofilms were grown in LB medium at 37 °C for 16 h. After removing the LB medium containing the planktonic populations of bacteria, the biofilms were washed with sterilized PBS three times, and the medium containing serial concentrations of AMPs was added. After a 16 h incubation at 37 °C, survival bacteria inside the biofilm were detected by plating on LB medium. For observation by confocal microscopy, the bacteria were transformed with pPABYm3-GFPuv. Bacterial biofilms were grown in 24-well plates with round glass coverslips. The biofilms with or without treatment with AMPs were observed using a confocal microscope (Leica TCS SP5).

### Statistical analysis

To analyze the statistical significance of differences between more than two sets of data, 1-way analysis of variance followed by Bonferroni multiple-comparison test was assessed. For analyzing nonparametrically distributed data, a Mann–Whitney U-test was used. Survival curves were compared with log-rank tests. A *P* < 0.05 was considered statistically significant.

## Supplementary Information

Supplementary Information
