# Supplementary material for: Identification of potential therapeutic antimicrobial peptides against Acinetobacter baumannii in a mouse model of pneumonia
Source: Sci Rep. 2021 Apr 1;11:7318. doi: 10.1038/s41598-021-86844-5 (PMC8016998; doi:10.1038/s41598-021-86844-5)
Supplement: Supplementary file 1 — Supplementary Information [file 41598_2021_86844_MOESM1_ESM.pdf]

Identification of potential therapeutic antimicrobial peptides against *Acinetobacter baumannii* in a mouse model of pneumonia

Chiau-Jing Jung[a, *], You-Di Liao[b], Chih-Chieh Hsu[c], Ting-Yu Huang[d], Yu-Chung Chuang[e], Jeng-Wei Chen[f, g], Yu-Min Kuo[g] and Jean-San Chia[d, h, *]

[a]Department of Microbiology and Immunology, School of Medicine, College of Medicine, Taipei Medical University, Taipei, Taiwan
[b]Institute of Biomedical Sciences, Academia Sinica, Taipei, Taiwan
[c]Graduate Institute of Oral Biology, School of Dentistry, National Taiwan University, Taipei, Taiwan,
[d]Graduate Institute of Microbiology, College of Medicine, National Taiwan University, Taipei, Taiwan
[e]Department of Internal Medicine, National Taiwan University Hospital, Taipei, Taiwan
[f]Division of Cardiovascular Surgery, Department of Surgery, National Taiwan University Hospital, Taipei, Taiwan
[g]Graduate institute of clinical medicine, College of Medicine, National Taiwan University, Taipei, Taiwan
[h]Graduate Institute of Clinical Dentistry, School of Dentistry, National Taiwan University, Taipei, Taiwan

***Co-corresponding authors:**
Jean-San Chia
Department of Microbiology, College of Medicine, National Taiwan University,
No. 1, Jen Ai Road Section 1, Taipei, 10051, Taiwan
Tel: 886-2-23123456 ext. 88222;
E-mail: chiajs@ntu.edu.tw
Chiau-Jing Jung
Department of Microbiology and Immunology, School of Medicine, College of Medicine, Taipei Medical University,
No. 250, Wuxing Street, Taipei 11031, Taiwan
Tel: 886-2-2736-1661 ext. 7159
E-mail: cjjung@tmu.edu.tw

Supplementary Figure S1

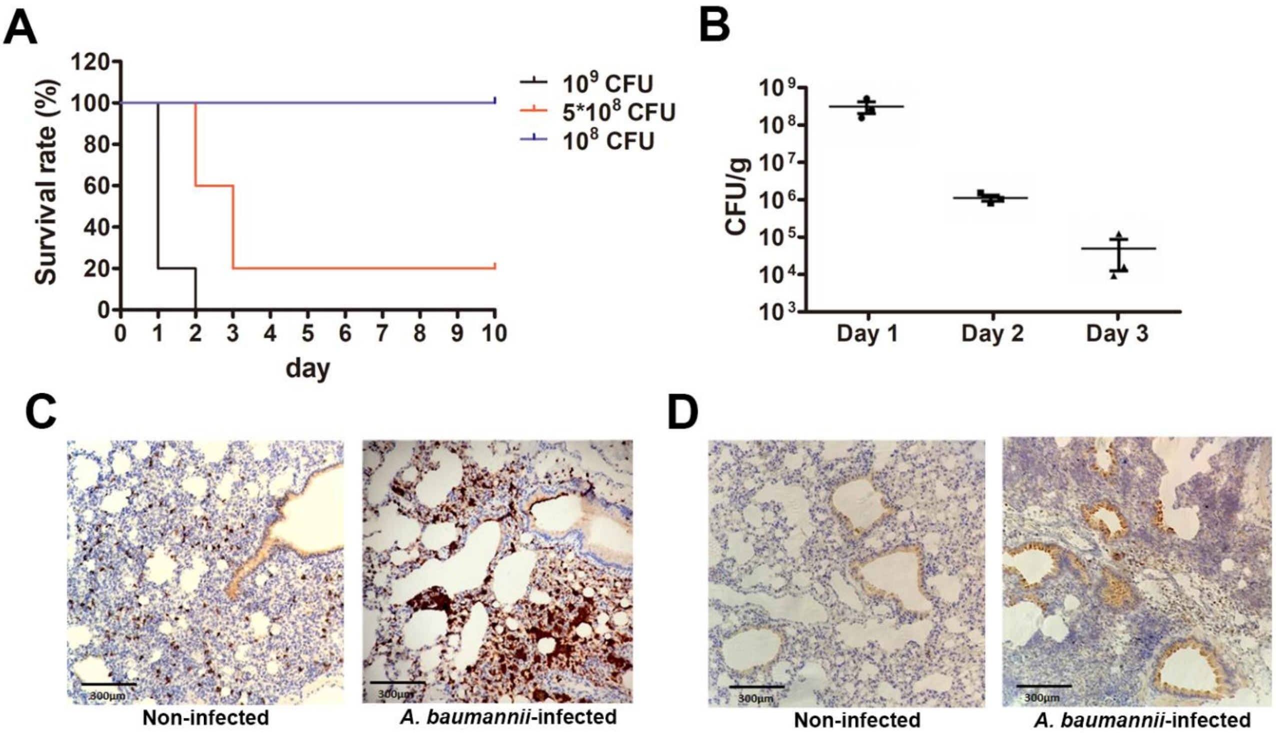

Supplementary Figure S1 Experimental *A. baumannii* pneumonia mouse model

A. Survival of mice with intratracheal *A. baumannii* ATCC 17978 infection at doses of 1 × 10^9, 5 × 10^8, and 1 × 10^8 CFU. Mouse survival was monitored daily (n = 5/group).

B. Bacterial colonization inside the lung was detected. Mice were intratracheally infected with *A. baumannii* ATCC 17978 at a dose of 1 × 10^8 CFU. Lung tissue was harvested 1, 2, or 3 d after infection. After homogenization by sonication, the bacteria that had colonized inside the lung tissue was quantified by plating on LB agar plates. Each point represents one mouse.

C. and D. Immunohistochemical analysis of *A. baumannii*-infected lung tissue. Mice were intratracheally infected with *A. baumannii* ATCC 17978 at a dose of 1 × 10^8 CFU. Infiltration of neutrophils (C) and macrophages (D) was detected by anti-Ly6G and anti-F4/80 antibodies one day after infection, respectively.

Supplementary Figure S2

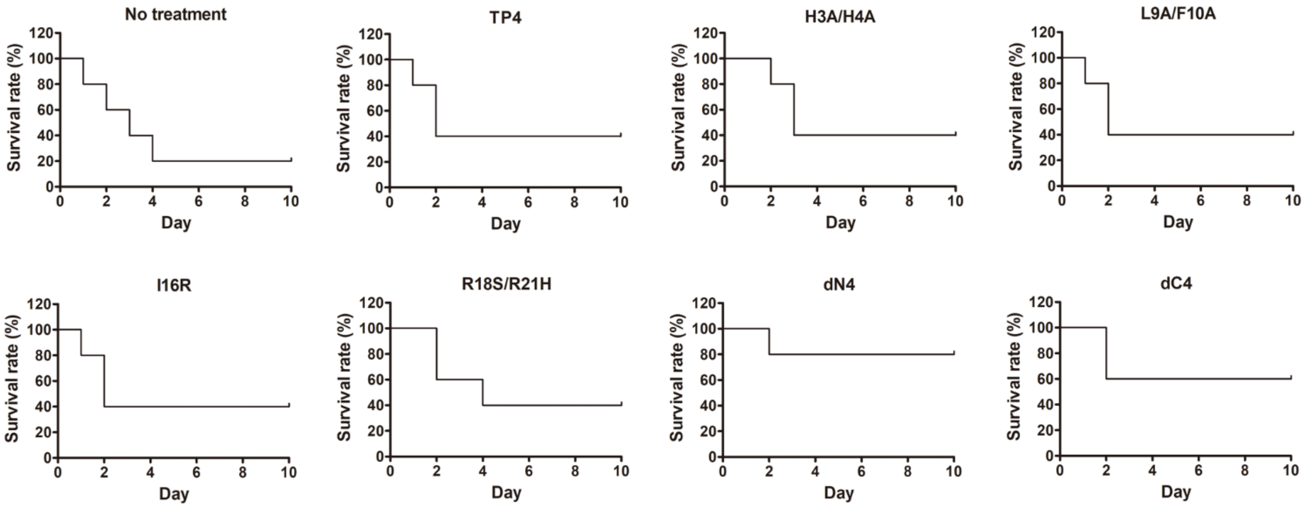

Supplementary Figure S2 Peritoneal administration of TP4 derivatives in *A. baumannii*-induced pneumonia mouse models

Mice were intratracheally infected with *A. baumannii* ATCC 17978 at a dose of 5×10^8 CFU. TP4 derivatives (2 mg/kg) were peritoneally administrated 4 h after bacterial infection, and mouse survival was detected daily (n = 5/group).

Supplementary Table 1 The structures and antimicrobial activity against *Acinetobacter baumannii* of 58 AMPs

The antimicrobial peptides with antimicrobial activity[a]		
AMPs	sequence	reference
GW-A2	GAKYAKIIYNYLKKIANALW	*Int. J Antimicrob Agent.*2008;32:130-138.
BMAP-27	GRFKRFRKKFKKLFKKLSPVIPLLHLG	*J Biol Chem.* 1996;271:28375-81.
CAME	KWKLFKKIGIGAVLKVLTTG-NH_2	*Antimicrob Agents Chemother.* 014;58(3):1622-9.
SMAP-29	RGLRRLGRKIAHGVKKYGPTVLRIIRIAG-NH_2	*Antimicrob. Agents Chemother.* 2001; 45:331–334.
GW-H1a	GYNYAKKLANLAKKFANALW-NH_2	*Int. J Antimicrob Agent.* 2008;32:130-138.
Latarcin-2a	GLFGKLIKKFGRKAISYAVKKARGKH	*J Biol Chem.* 2006;281:20983-92.
Maximin H2	ILGPVLSMVGSALGGLIKKI-NH_2	*Peptides.* 2002;23:427-35.
NRC12	GWKKWFNRAKKVGKTVGGLAVDHYL-NH_2	*Antimicrob Agents Chemother.* 2003;47:2464-70.
Pilosulin	GLGSVFGRLARILGRVIPKV-NH_2	*Arch Biochem Biophys.* 2005;434:358-64.
Pleurocidin	GWGSFFKKAAHVGKHVGKAALTHYL-NH_2	*Peptides.* 2011;32(8):1748-54.
GW-Q6	GIKIAKKAITIAKKIAKIYW	*Int. J Antimicrob Agent 2008;*32;130-138.
TP4	FIHHIIGGLFSAGKAIHRLIRRRRR	*PloS one.* 2012;7(11); e50263
The antimicrobial peptides without antimicrobial activity[a]		
1029	KQFRIRVRV	*Antimicrob. Agrnts Chemother.* 2012; 56:2696-2704
1037	KRFRIRVRV	*Antimicrob. Agrnts Chemother.* 2012; 56:2696-2704
Buforin-2	TRSSRAGLQFPVGRVHRLLRK	*Biochem Biophys Res Commun.* 1996; 218:408-13.
CAMA	TRSSRAGLQFPVGRVHRLLRK	*Antimicrob Agents Chemother.* 2014;58(3):1622-9.
CRAMP	GLLRKGGEKIGEKLKKIGQKIKNFFQKLVPQPEQ	*J Biol Chem.* 1997 ;272(20):13088-93.
HPMA	AKKVFKRLG IGKFLHSAKKF-NH_2	*Antimicrob Agents Chemother.* 2014;58(3):1622-9.
HPME	AKKVFKRLGIGAVLKVLTTG	*Antimicrob Agents Chemother.* 2014;58(3):1622-9.

KRA	KRAKKFFKKLK-NH_2	*BMC-Microbiology*. 2011; **11**:114.
Latarcin-1	SMWSGMWRRKLKKLRNALKKKLKGE	*J Biol Chem.* 2006; 281:20983-92.
LL37	LLGDFFRKSKEKIGKEFKRIVQRIKDFLRNLVPRTES	*J Invest Dermatol.* 120;810-816.
Maximn 3	GIGGKILSGLKTAKGAAKELASTYLH	*Peptides.* 2002; 23:427-35.
Nigrocin (mutant)	GLLSGILGAGKHIVCGLSGLK	*Mol Cell Proteomics.* 2007;6:882-94.
NRC-13	GWRTLLKKAEVKTVGKLALKHYL	*Antimicrob Agents Chemother.*2003; 47:2464-2470
Odorranain-B1	AALKGCWTKSIPPKPCFGKR	*Mol Cell Proteomics*. 2007;6:882-94.
OdP1a (mutant)	VIPFVASVAAEMMQHVYCAASKKC	*Mol Cell Proteomics.* 2007;6:882-94.
P318	KIGEKLKKIGQKIKNFFQKLVPQPEQ	*Antimicrob Agents Chemother.* 2014;58:5395-404.
PAM2	RPWAGNGSVHRYTVLSPRLKTQ	*PLoS One.* 2011;6:e24030
Paracentrin 1	EVASFDKSKLK	*AMB Express* 2014; 4:78
Para 1 (mutant)	KVAWFDKSKLK-NH_2	Synthesis
Para 2 (mutant)	Ac-LVAWFDKSKLK-NH_2	Synthesis
Para 3 (mutant)	Ac-LVWSFKKSKLK-NH_2	Synthesis
T9F	H-RFRRLRKKFRKRLKKI-NH_2	*PloS one.* 2014; 9(12):e114605.
T9W	H-RFRRLRKKWRKRLKKI-NH_2	*PloS one. 2014; 9(12):e114605.*
Thanatin	GSKKPVPIIYCNRRTGKCQRM	*Proc Natl Acad Sci U S A.* 1996; 93:1221-5.
Tn-AFP1	LMCTHPLDCSN	*Peptides.* 2011;32(8):1741-7.
TP359	H-MYRKKALK-NH_2	*BMC Microbiol.* 2016;16(1):192.
β-amyloid peptide 1-42	H-DAEFRHDSGYEVHHQKLVFFAEDVGSNKGAIIGLMVGGVVI-OH	*Sci Rep.* 2016;6:32228.
Clavanin-4	VFQFLGKIIRRVGNFVRGFSRVF	*Infect Immun.* 1997. 2898–2903
Clavanin-8	VFQFLVKIIRRVVRFVRRFSRVF	*Infect Immun.* 1997. 2898–2903
ECP (5-17P22-36)	FTRAQWFAIQHISPRCTIAMRAINNYRWR	*Antimicrob Agents Chemother.* 2016;60(10):6313-25.
GE33	GFFALIPKIISSPLFKTLLSAVGSALSSSGGQE	*FEBS Lett.* 1988; 242: 161-166

Indolicidin	ILPWKWWPWWPWRR-NH_2	*Biochim Biophys Acta.* 2006;1758(10):1596-608
Leu10	GEAFSAGVHRLANG-NH_2	*ACS Comb Sci.* 2015;17:156-63.
GW-A1	GAKYAKYIYNFYKYIAKYIW	*Int J Antimicrob Agents.* 2008;32(2):130-8.
GW-A4	GAKALTKAATAFTKFYKTIW	*Int J Antimicrob Agents.* 2008;32(2):130-8.
GW-A5	GATYAKKIIKTITKIATTAW	*Int J Antimicrob Agents.* 2008;32(2):130-8.
GW-M1	GANAAKKLATFAKKIFTAYW	*Int J Antimicrob Agents*. 2008;32(2):130-8.
GW-M2a	GANAAKKFATIAKKFINYLW	*Int J Antimicrob Agents.* 2008;32(2):130-8.
GW-M4	GYKYINNIIKYINKFFKYIW	*Int J Antimicrob Agents.* 2008;32(2):130-8.
GW-Q3	GANLAKKFYTYINKFINYAW	*Int J Antimicrob Agents.* 2008;32(2):130-8.
GL13-NH2	GQIINLKASLDLL-($CONH_2$)	*Peptides.* 2008;29(12):2118-27.
GL13-D/N-NH2	GQIINLKASLNLL-($CONH_2$)	*Peptides.* 2008;29(12):2118-27.
Lfcin B11	RRWQWRMKKLG	*J Dairy Sci.* 2013;96(12):7511-20.
Lfcin B25	FKCRRWQWRMKKLGAPSITCVRRAF	*J Dairy Sci.* 2013;96(12):7511-20.
Lfcin B6	RRWQWR	*J Dairy Sci.* 2013;96(12):7511-20.
LL-32	LLGRFFRKLIRKILKRFKRIVQRILRFLRNLV	*J Biol Chem.* 2008;283:32637-43.

a. The antimicrobial activity against *Acinetobacter baumannii* were determined by a inhibition zone assay, performed as previous described[1]. The AMPs with an inhibition zone diameter ≥ 10 mm were determined as the AMPs with antimicrobial activity. Their MIC and MBC of the AMPs with antimicrobial activity were further determined in this study.

Supplementary Table 2 Minimal inhibitory concentration (MIC) and minimal bactericidal concentration (MBC) of antimicrobial peptides against clinical multiple drug-resistant (MDR) *Acinetobacter baumannii* strains

	J49		K2		Y92		W11		U80		U95		O59		D29	
Peptide (µg/ml)	MIC	MBC	MIC	MBC	MIC	MBC	MIC	MBC	MIC	MBC	MIC	MBC	MIC	MBC	MIC	MBC
GW-A2	16	16	32	64	16	16	16	16	16	16	32	64	32	32	16	16
BMAP-27	8	8	16	32	8	8	16	16	8	16	16	16	16	16	16	16
CAME	16	32	8	16	32	32	16	32	16	32	32	64	32	64	32	32
SMAP-29	32	64	16	32	8	8	8	8	8	8	8	16	8	16	8	8
GW-H1a	8	8	16	16	8	8	16	16	16	32	16	32	16	32	16	32
Latarcin 2a	64	64	64	64	8	8	8	8	16	16	16	16	16	16	8	16
Maximin H2	16	16	16	32	64	128	64	128	128	128	128	128	128	128	128	128
NRC12	16	16	16	32	16	16	16	16	32	32	32	32	32	32	16	16
Pilosulin	16	16	16	32	8	8	16	16	16	16	32	32	16	16	16	32
Pleurocidin	16	16	16	32	8	8	16	16	16	16	32	32	16	16	16	32
GW-Q6	16	16	8	8	8	8	16	16	16	16	16	32	16	32	16	32
TP4	16	16	16	32	16	16	16	16	32	32	32	64	32	32	32	32

	L87		AO80		AS40		AK29		AK61		AE5		AE6		AF11	
Peptide (µg/ml)	MIC	MBC	MIC	MBC	MIC	MBC	MIC	MBC	MIC	MBC	MIC	MBC	MIC	MBC	MIC	MBC
GW-A2	16	16	8	16	8	16	8	8	8	8	16	16	16	32	16	32
BMAP-27	16	16	8	8	8	8	4	8	8	8	8	16	16	16	8	16
CAME	32	64	8	16	8	8	8	16	8	16	32	32	32	32	16	32
SMAP-29	16	16	4	8	8	8	4	8	4	8	8	8	8	16	8	8
GW-H1a	16	32	8	8	8	8	8	8	8	8	16	16	8	16	8	16
Latarcin 2a	16	16	8	8	8	8	8	8	8	8	8	8	8	8	8	8
Maximin H2	128	128	64	128	64	64	32	32	64	64	64	128	128	128	128	128
NRC12	32	32	32	32	32	32	8	8	32	32	16	16	16	16	16	16
Pilosulin	16	16	16	32	16	16	16	16	16	16	8	16	16	16	8	16
Pleurocidin	32	32	8	8	8	8	8	16	16	16	16	16	16	16	8	16
GW-Q6	16	16	16	16	16	16	8	8	16	16	8	8	8	8	8	8
TP4	32	32	32	32	32	32	16	16	32	32	16	32	16	16	16	16

	AF12		AF93		AG51		AN10		AL87		AZ93	
Peptide (µg/ml)	MIC	MBC	MIC	MBC	MIC	MBC	MIC	MBC	MIC	MBC	MIC	MBC
GW-A2	8	8	16	16	8	8	8	8	8	8	8	16
BMAP-27	8	8	8	8	4	8	4	4	8	8	8	8
CAME	16	16	32	32	16	32	16	32	32	32	8	8
SMAP-29	4	4	8	8	4	4	4	4	8	8	8	8
GW-H1a	8	16	8	8	8	8	8	8	8	8	8	8
Latarcin 2a	8	8	8	8	4	4	4	4	4	4	8	8
Maximin H2	64	64	64	64	32	64	64	64	64	64	64	64
NRC12	8	16	16	16	8	8	8	8	8	8	32	32
Pilosulin	8	16	8	8	8	16	8	8	8	8	16	16
Pleurocidin	8	8	8	16	8	8	16	16	8	8	8	8
QW-Q6	8	8	8	16	8	8	8	8	8	8	8	8
TP4	16	16	16	16	8	16	8	8	16	16	16	16

Supplementary Table 3 The antibiotic resistance of MDRAB clinical strains.

The antimicrobial susceptibilities of MDRAB clinical strains were determined with the VITEK 2 system.

	J49	K2	Y92	W11	U80	U95	O59	D29
Ceftazidime	R	R	R	R	R	R	R	R
Ciprofloxacin	R	-	R	R	R	R	R	R
Cefepime	R	R	R	R	R	R	R	R
Gentamicin	S	R	R	R	R	R	R	R
Imipenem	R	-	R	R	R	R	R	R
Levofloxacin	R	R	R	R	R	R	R	R
Meropenem	R	-	R	R	R	R	R	R
Ampicillin/Sulbactam	I	-	R	R	R	R	R	R
Piperacillin/Tazobactam	R	R	R	R	R	R	R	R

	L87	AO80	AS40	AK29	AK61	AE5	AE6	AF11
Ceftazidime	R	R	R	R	R	R	R	R
Ciprofloxacin	R	R	R	R	R	R	R	R
Cefepime	R	R	R	R	R	R	R	R
Gentamicin	R	R	R	R	R	R	R	R
Imipenem	R	R	R	R	R	R	R	R
Levofloxacin	R	R	I	R	R	R	R	R
Meropenem	R	R	R	R	R	R	R	R
Ampicillin/Sulbactam	R	R	R	R	R	R	R	R
Piperacillin/Tazobactam	R	R	R	R	R	R	R	R

	AF12	AF93	AG51	AN10	AL87	AZ93	BF50	MO91
Ceftazidime	R	R	R	R	R	R	R	R
Ciprofloxacin	R	R	R	R	R	R	R	R
Cefepime	R	R	R	R	R	R	R	R
Gentamicin	R	R	R	R	R	R	R	R
Imipenem	R	R	R	R	R	R	S	R
Levofloxacin	R	R	R	R	R	R	R	R
Meropenem	R	R	R	R	R	R	S	R
Ampicillin/Sulbactam	R	R	I	R	R	R	R	R
Piperacillin/Tazobactam	R	R	R	R	R	R	R	R

Supplementary Table 4 Minimal inhibitory concentration (MIC) and minimal bactericidal concentration (MBC) of dN4 and dC4 against clinical multiple drug-resistant (MDR) *Acinetobacter baumannii* strains in the presence of plasma components.

		ATCC strain				MDRAB strain			
		17978		19606		BF50		MO91	
Peptide (μg/ml)	10% plasma	MIC	MBC	MIC	MBC	MIC	MBC	MIC	MBC
dN4	-	32	32	16	64	16	64	32	32
	+	64	128	16	128	16	128	64	64
dC4	-	32	32	32	32	32	32	32	32
	+	64	128	64	64	64	128	64	64

Reference

1 Wang, S. H., Tang, T. W., Wu, E., Wang, D. W. & Liao, Y. D. Anionic surfactant-facilitated coating of antimicrobial peptide and antibiotic reduces biomaterial-associated infection. *ACS Biomater Sci Eng* **6**, 4561-4572, doi:10.1021/acsbiomaterials.0c00716 (2020).
